# Saliva Exhibits High Sensitivity and Specificity for the Detection of SARS-COV-2

**DOI:** 10.3390/diseases9020038

**Published:** 2021-05-20

**Authors:** Ibrahim Warsi, Zohaib Khurshid, Hamda Shazam, Muhammad Farooq Umer, Eisha Imran, Muhammad Owais Khan, Paul Desmond Slowey, J. Max Goodson

**Affiliations:** 1 Medical Sciences in Clinical Investigation, Harvard Medical School, Boston, MA 02115, USA; ahmed_warsi@hms.harvard.edu; 2 The Forsyth Institute, Cambridge, MA 02142, USA; mgoodson@forsyth.org; 3 Department of Prosthodontics and Dental Implantology, College of Dentistry, King Faisal University, Al-Ahsa 31982, Saudi Arabia; 4 Department of Oral Pathology, College of Dentistry, Ziauddin University, Karachi 74700, Pakistan; hamdashazam@gmail.com; 5 Al-Shifa School of Public Health, Al-Shifa Trust, Jhelum Road, Rawalpindi, Punjab 46000, Pakistan; rafooq@hotmail.com; 6 Department of Dental Materials, Institute of Medical Sciences, HITEC Dental College, Taxilla 751010, Pakistan; eishaimran@ymail.com; 7 Dow International Dental College (DIDC), Dow University of Health Sciences, Karachi 74200, Pakistan; owais.1@me.com; 8 Health Science Center, School of Public Health, Xi’an Jiaotong University, Xi’an 710061, China; pds@4saliva.com; 9 Oasis Diagnostics® Corporation, Vancouver, WA 98686, USA; 10 Department of Oral Medicine, Infection, and Immunology, Harvard School of Dental Medicine, Boston, MA 02115, USA

**Keywords:** saliva, COVID-19, SARS-CoV-2, infection, diagnosis, polymerase chain reaction

## Abstract

In the wake of the COVID-19 pandemic, it is crucial to assess the application of a multitude of effective diagnostic specimens for conducting mass testing, for accurate diagnosis and to formulate strategies for its prevention and control. As one of the most versatile and amenable specimen options, saliva offers great advantages for widespread screening strategies due to its non-invasive properties, cost-effectiveness, excellent stability and minimal risk of cross-infection. This review attempts to outline the scientific rationale for detection of SARS-COV-2 in saliva specimens. By combining the data obtained from ten chosen published clinical studies, we calculated the pooled sensitivity and specificity using an online calculator. Through evidence, we established that SARS-COV-2 is detectable in saliva with a high degree of diagnostic sensitivity (87%) and specificity (98%). We also presented a review of emerging technologies approved by the FDA for detection of SARS-COV-2 in oral fluids (saliva and sputum) using polymerase chain reaction methods. Given the challenges involved in obtaining invasive specimens from the naso- and oropharynx, saliva can serve as an easy to collect diagnostic specimen for screening in the work environment, schools and for home testing. Furthermore, saliva offers the opportunity to screen early cases that can be missed by invasive sampling.

## 1. Introduction

**Figure 1:**
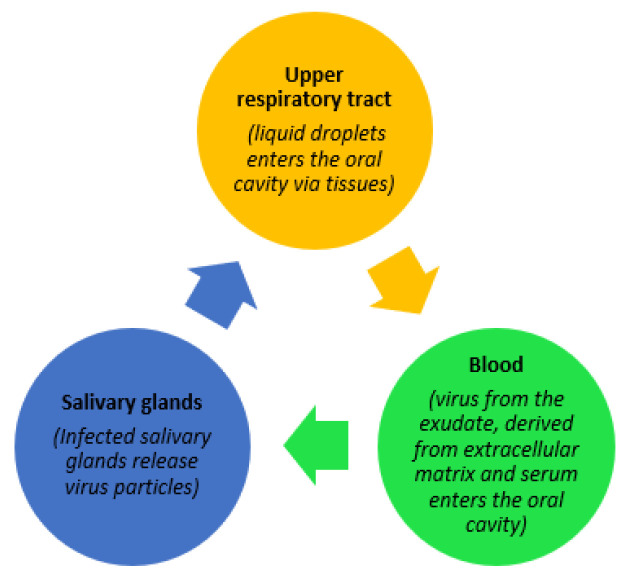
Possible trajectories for the presence of SARS-CoV-2 in saliva.

There is little debate that one of the major global healthcare challenges facing the world today is the coronavirus pandemic. The virus has been named Severe Acute Respiratory Syndrome Coronavirus-2 (SARS-CoV-2) [1]. This particular coronavirus (SARS-CoV-2) belongs to the family of beta coronaviruses, sharing many similarities with prior viral diseases such as SARS and MERS. Compared to these other viral infections, the current SARS-CoV-2 virus has a greater affinity for binding with ACE-2 receptors on host cell surfaces, which increases the contagious potential of the infection, associated with the virus, causing coronavirus disease 2019 (COVID-19) [2]. SARS-CoV-2 can be transmitted via direct or indirect contact. One of the primary sources of transmission of coronavirus is through salivary aerosols emitted from coughing, breathing, and even during speaking [2]. Figure 1, below, illustrates three potential trajectories for the presence of the virus in saliva as explained by Sabino-Silva et al. [3]. Contemporary studies have gathered evidence demonstrating molecular strategies adopted by the SARS-CoV-2 virus, enabling it to enter the host cell, causing a high rate of infectivity. Possible activation of the SARS-CoV-2 virus by gene expression of furin in salivary glands is also a noteworthy finding explained by Shang et al. [4]. Furin is typically expressed by salivary glands and its components are responsible for the regulation of different specific proteins while the gene itself is believed to cleave different viral toxins including coronaviruses. As a result, the severity of COVID-19 disease is increased if salivary infection is withdrawn from the salivary glands, while the presence of furin in saliva leads to a rapid progression of the disease through salivary droplets [5].

Since mid-December 2019, global morbidity associated with COVID-19 has surpassed 119 million, contributing to more than 2.6 million global deaths [6]. In the United States alone 29 million people have been affected, resulting in nearly 528,456 deaths [6]. As in the case of many other infectious diseases, early diagnosis of coronavirus and effective prevention strategies can help control COVID-19 transmission and limit deaths. To respond effectively to the COVID-19 pandemic, mass community testing will be required in multiple testing environments and this has recently been recognized by the new Biden Administration in the US who have allocated USD 50 billion to expand testing and laboratory capabilities. Rapid and accurate diagnosis of positive cases and contact management along with appropriate clinical management efforts and infection control will be critical components of the effort to fight the pandemic, in addition to the implementation of community mitigation efforts [7]. Integral to all of these efforts is the implementation of widespread testing efforts to test as many members of the public likely to be infected. While testing protocols were initially limited to oropharyngeal and nasopharyngeal swab-based methods, and reverse transcription polymerase chain reaction (RT-PCR) tests, these limited options make it difficult to effectively reach all sectors of the community. This is in addition to an early shortage of funding, as well as the lack of availability of test kits and personal protective equipment (PPE) for healthcare professionals carrying out the tests, and most importantly a global shortage of nasopharyngeal and oropharyngeal swabs, which led to the evaluation of alternate specimen types. The combination of these unexpected circumstances provided an ideal set up for the evaluation and validation for inexpensive, rapid, easy-to-handle, and non-invasive methodologies, for instance saliva, which could be integrated with existing testing methods (RT-PCR, PCR) seamlessly while still meeting the stringent performance needed in terms of high degrees of sensitivity and specificity [8].

## 2. Regulatory Framework and Introduction of Salivary Diagnostics for COVID-19

In the United States, the Centers for Disease Control and Prevention (CDC) works closely with the Federal Drug Administration (FDA) who are responsible for the issuance of an interim regulatory approval, known as the Emergency Use Authorization (EUA), which companies working in the COVID-19 testing area are required to obtain before commercializing their specific tests. The FDA is also responsible for the issuance of guidance documents to provide policy and a framework for laboratories and commercial manufacturers to accelerate the availability of alternate novel coronavirus (COVID-19) tests [9,10]. Following the CDC and FDA’s policy guidelines, the Rutgers Clinical Genomics Laboratory and Infinite Biologics, using the Thermo Fisher TaqPath™ SARS-CoV-2 assay for high throughput testing, obtained EUA approval in April 2020 enabling them to begin commercializing their salivary test for SARS-CoV-2 [11]. Table 1 shows a timeline of the salivary testing kits.

The assay is based on a real-time reverse transcription polymerase chain reaction (rRT-PCR) test, performed in a laboratory. The study carried out by Rutgers in support of their EUA approval indicated a one hundred percent positive and negative agreement for SARS-COV-2 detection in saliva compared to oropharyngeal and nasopharyngeal swab tests [11]. These findings were validated by the New Jersey State Health Department using a previously authorized CDC SARS-COV-2, RT-PCR diagnostic panel [11]. As of today, multiple studies have investigated the diagnostic potential of oral fluids (sputum and salivary) for COVID-19, using various methods including real-time reverse transcription polymerase chain reaction and real-time loop-mediated isothermal amplification methods [10], each demonstrating a high degree of positive and negative agreement in comparison to the conventional CDC-approved RT-PCR test. Since the approval of the Rutgers test, multiple saliva tests have received FDA EUAs. A selection of the most important tests are illustrated in Table 2.

## 3. Review of Studies on Salivary Diagnostics

The literature published up to 22 July 2020 investigating the presence of SARS-CoV-2 RNA in saliva was scrutinized and included in this review. Table 2 describes assays that have received FDA EUA approval containing more recent data. The published material included in this review targeted case reports and series, cross-sectional studies and observational studies. Different approaches and collection techniques were used in the trials included here, such as collection of saliva by cough, passive collection from posterior oro-pharynx, simple swab or a whole saliva collection technique. As of 22 July 2020, RT-PCR using nasopharyngeal respiratory specimens is very much the gold standard for the qualitative detection of the SARS-COV-2 virus.

A study in Hong Kong is the earliest available reported study during the course of pandemic, which investigated the presence of SARS-CoV-2 in saliva in 11 COVID-19-positive patients. The patients were tested at various phases including during their recovery phase and, at that time, a decline in salivary SARS-CoV-2 RNA was observed [24]. Early evidence from Wuhan (China), revealed that in a cohort of 16 COVID-19 patients, the SARS-CoV-2 viral titers were discovered using oral swabs, anal swabs and in plasma; but investigators also found that the detection overlap between the three samples was not consistent. Further investigations revealed positive oral swab results in eight patients following medical treatment [20]. Important studies evaluated the range of viral loads present in saliva and researchers found values ranging from 9.9 × 10^2^ to 1.2 × 10^8^ copies/mL [17,25,26,27,28,29,30,31,32]. Other investigators evaluated and compared the difference in efficiency of saliva using oro-nasopharyngeal swabs for the detection of viral load. Other studies validated the sensitivity of saliva samples versus nasopharyngeal swabs using RT-qPCR analysis and these are reported in multiple studies [12,21,30,32,33,34,35,36,37]. In a case study on a COVID-19-infected neonate, Korean investigators identified that serial sampling of saliva demonstrated a reduction in viral load over a 27-day follow-up [15]. Historically, perhaps, the first study that investigated the clinical progression of the disease course with temporal viral load also confirmed that posterior oropharyngeal salivary viral load was highest in the first week after symptom onset, and subsequently declined with time [17]. In a separate study, investigators from the USA compared the sensitivity and specificity of nasopharyngeal swabs versus saliva samples for the detection of SARS-CoV-2 via RT-PCR and demonstrated that saliva had a higher detection sensitivity, maintained consistency throughout the course of infection, and demonstrated less variability during the self-sampling collection process [18]. Furthermore, in a case report of two patients from an Italian study, investigators demonstrated the positive detection of SARS-COV-2 virus in saliva specimens, while respiratory swab specimens indicated a negative result in both cases [13]. In a study by Wong et al., the cost of these two specimens was compared and it was estimated that saliva specimens (USD 8.24 per 100) were much more economical when compared to the use of NPS (USD 104.87 per 100) [38]. McCormick et al. introduced a technology described as a “faster” and “novel” technique for the diagnosis of COVID-19 in saliva using the Xpert Xpress technology [39] (Cepheid Diagnostics) while Chen et al., in another qualitative study, compared specific differences in sensitivity between the two specimens using the same Xpert-Xpress rapid real time RT-PCR test and concluded that there was no significant difference in detection rate [40]. Nagura et al. evaluated the sensitivity of samples using different techniques for analysis of saliva samples and demonstrated that rapid antigen tests exhibited lower sensitivity than other approaches, particularly RT-qPCR, direct RT-qPCR and RT-LAMP techniques [35]. These results echoed the findings of Mak et al., demonstrating lower sensitivity in the case of antigen tests [41]. The presence of SARS-CoV-2 RNA in the saliva of asymptomatic patients [35] was a noteworthy finding in a study by Nagura et al., whose results were in consensus with Bosworth et al., who identified early positive saliva results in healthcare workers who were later found to be symptomatic for COVID-19 [42].

In two additional studies, the variability of specimen collection time was evaluated. Hung et al. in their work detected a higher viral load early in the morning in contrast to nighttime [43], while Tajima et al. observed that samples taken during the day were less likely to be in harmony with results collected in the early morning [36]. Another study from Wuhan (China), demonstrated that detectable levels of ACE-2 protein are expressed in the salivary glands. In this experimental work, the authors demonstrated that in the direct collection of saliva from salivary orifices, the SARS-COV-2 virus can be detected in only 4 out of 31 COVID-19 patients, who were critically ill patients [14], and this does not seem to be typical. Other studies have also been carried out by sampling sputum, but in this sample matrix, the detection of SARS-COV-2 is quite low (refer to Table 2). In contrast, pooled or saliva obtained by the drooling method (the recognized gold standard method), demonstrates the highest sensitivity for the detection of viral load [44]. In a cohort of 25 COVID-19 patients, the Italian investigators demonstrated a 100% detection sensitivity using pooled whole salivary samples [12].

## 4. Sensitivity and Specificity of Salivary Diagnostics for SARS-CoV-2 Testing

Table 1 highlights a selection of ten studies comparing the sensitivity and specificity of saliva with conventional methods of specimen sampling (nasopharyngeal, oropharyngeal and bronchoalveolar lavage) in detecting SARS-COV-2, for the diagnosis of COVID-19, using the recognized RT-PCR method. Limited studies have demonstrated the comparability or superiority of saliva sampling, relative to conventional swab-based sampling; however, the results for saliva are compelling. As mentioned earlier, we were able to compile the results from current and previously reported studies and have been able to calculate a pooled sensitivity and specificity for saliva specimen collection in the detection of SARS-CoV-2. The table below (see Table 2) demonstrates a pooled sensitivity of approximately 87% and a specificity of 98%. From the combined results, the probability of a positive test result being a true positive (PPV/True-positive) is 98%, and the probability of a negative test result being a true negative result (NPV/False-positive) is 86%. These findings indicate that salivary-based detection of SARS-CoV-2 using the RT-PCR method has a high diagnostic accuracy for positive cases but may lack accuracy for the detection of false-positive cases. However, these performance data mirror that of data obtained using nasopharyngeal swabs and represents a solid alternative for diagnostic purposes.

### 4.1. Strengths and Limitations of Salivary Diagnostics

Currently, recommended specimens for use in RT-PCR diagnostic testing for COVID-19 infection include nasal, oropharyngeal and blood samples. In the case of lower respiratory tract infections, expectorated sputum specimens are also considered [45,46]. Use of these specimens for the diagnosis of COVID-19 can introduce a few limitations. Bearing in mind that there is close contact between COVID-19 patients and healthcare workers collecting patient specimens, the exposure risk of healthcare workers to the virus is enhanced using such specimens. Furthermore, the shortage of skilled staff, personal protective equipment (PPE), testing kits and reagents are factors that limit the testing capacity during a pandemic crisis. Given the invasive nature of sampling, subjects also experience significant discomfort during the collection process, increasing the risk of injury and reducing patient compliance. A number of contraindications of NPS including nasal septum deviation and coagulopathy have also been reported, further signifying the need for additional non-invasive and simple diagnostic approaches [47]. It is known that oral fluid (sputum and saliva) obtained by coughing demonstrates a higher detection sensitivity for SARS-COV-2, compared to sputum or saliva alone [16,48], but it has also been observed that patient compliance during collection of an oral fluid specimen by coughing alone is very low, making it an unreliable diagnostic specimen [48]. It is important to point out that the conventional use of oropharyngeal and nasopharyngeal swab specimens leads to a significant proportion of false-negative findings when compared to the application of saliva specimens [2,44]. It is still unclear whether swab specimens can accurately reflect viral titers in the individual or reflect disease progression.

Over the past few decades, saliva has become a highly viable diagnostic tool for the detection of various oral and systemic diseases [49,50]. Human saliva is a complex mixture of cellular constituents, minor/major salivary gland secretions, proteinases, proteins and molecular organisms [51]. The discovery of numerous robust salivary biomarkers has also led to the detection of infectious diseases during early stages of disease [45,52,53].

The inclusion of saliva samples for the detection of SARS-CoV-2 coronavirus is a major step forward in the fight to identify patients suffering from the disease. Due to its easily accessible nature, it can be readily obtained from patients in a non-invasive fashion, thereby reducing the risk of nosocomial infections among healthcare workers [8,54]. Equally importantly, saliva samples possess high sensitivity and specificity when compared to nasopharyngeal swabs for the detection of SARS-CoV-2 coronavirus (refer to Table 2). Intriguingly, there are also a few studies that report that SARS-CoV-2 is detectable in saliva samples but not in nasopharyngeal swabs [13,18]. It is documented that saliva collection is beneficial in cases where screening of infected individuals is required on a larger scale such as in the community, in a drive-through setting, in a hospital setup or in locations with access to limited medical resources. In addition, it has also been previously documented that certain viral strains may survive in saliva for 29 days post-infection, enhancing the possibility of disease detection even at later stages of the disease [3].

Evidence generated so far in the literature indicates that higher sensitivity of SARS-CoV-2 detection using salivary diagnostics is dependent upon the type of sampling strategy and the diagnostic tests used (Table 2) [21,44]. In the collection of sputum or saliva, extra caution should be sought to avoid excessive contamination by oral and upper respiratory bacteria. This potential issue can be addressed by rinsing the oral cavity before sample collection and collecting the saliva in a sterile container used for sputum or urine collection [44].

### 4.2. Emerging Technologies in Salivary Diagnostics for COVID-19

Besides the CDC-approved RT-PCR test for the detection of SARS-COV-2, many other inexpensive, fast detection methods for mass screening purposes have been recently approved by the FDA under the EUA (Emergency Use Authorization) mechanism [10]. As illustrated in Table 2, the diagnostic specificity and sensitivity of oral fluids (saliva and sputum) are very high in controlled studies with smaller sample sizes. What is currently needed are larger-scale validation studies that could validate the implementation of these tests for mainstream diagnostic purposes. Most controlled studies have validated the use of salivary diagnostics in relatively small populations and it may be still too early to implement salivary diagnostics as the sole method for the detection of SARS-CoV-2 viral load, however, so far the results look promising. In mass screening programs, however, due to human error, the issue of obtaining a false negative result far outweighs the risk of having a false-positive result, so tests with high sensitivity are desirable. False-negative results may arise due to improper specimen collection, degradation of viral RNA during the logistical phase, the presence of RT-PCR inhibitors and the use of un-validated extraction methods or assay reagents. On the other hand, false-positive results may arise from cross-contamination of specimens or RNA contamination during the handling or preparation phase.

### 4.3. Direction for Future Studies

Large-scale prospective studies are needed to establish the temporal trends in salivary viral titers and tie them in with the course of infection or as markers of disease severity. Although preliminary evidence indicates that salivary detection of SARS-CoV-2 is feasible in mild or asymptomatic cases [16,17], once again this finding needs to be validated in a larger cohort. Large-scale, epidemiological studies are needed to compare the sensitivity and specificity of swab-based methods versus methods where saliva specimens are used. Here we note that there are differences in the literature where various types of oral samples collected have contributed to variability in the detection of SARS-CoV-2 (refer to Table 2). This in turn leads us to recommend that further studies should be performed to validate different oral fluid collection protocols and to compare viral detection rates [20,55]. Moreover, it is important to extend the application of salivary diagnostics to neglected or vulnerable populations such as pediatric populations, geriatrics and pregnant females. Generally speaking, the literature is unclear on whether SARS-CoV-2 detection is dependent on ACE-2 receptor expression at oral sites, versus nasopharyngeal sites, so we believe this relationship should be investigated in future studies [56]. To avoid the frequency of false-negative results in suspected positive cases, sampling a mix of multiple specimens (including oropharyngeal, nasopharyngeal, oral, sputum and saliva specimens) is recommended [57]. For proposed future research, we recommend the application of saliva for a number of studies aimed at disease detection and looking at progression. Saliva is an excellent matrix for the evaluation of salivary antibodies, so studies on a large cohort of individuals will provide advantages for monitoring disease progression in the COVID-19 area.

## 5. Conclusions

Saliva has shown true potential as an ideal non-invasive diagnostic specimen, with a high degree of sensitivity and specificity for the detection of the SARS-COV-2 virus. Saliva can also be used in the serial monitoring of viral load in COVID-19 patients. It is preferred in situations where nasopharyngeal and oropharyngeal swabs are difficult or should be avoided, and can certainly be used as a tool for self-collection. The significant potential for salivary diagnostics needs large-scale, longitudinal studies to confirm saliva’s role as a first-line diagnostic specimen for COVID-19 testing. Future studies should also corroborate early clinical manifestations of the COVID-19 infection with viral titers in saliva, to confirm the role of saliva in the early diagnosis and as an aid in limiting disease transmission.

## Figures and Tables

**Table 1:** Timeline of the saliva collection devices for the SARS-CoV-2 sampling during the pandemic.

Sr. No.	Tests	Date of Approval
1	MicroGenDX Laboratories obtains FDA EUA approval for 24-h saliva test [RT-PCR]	4 April 2020
2	Rutgers University/Infinite Biologics gets first FDA EUA Approval for saliva high throughput method connected to Thermo Fisher RT-PCR	13 April 2020
3	Curative receives FDA EUA for oral fluid test	19 April 2020
4	FDA announces changes in registration requirements	19 May 2020
5	MLB adopts saliva COVID-19 Testing	20 May 2020
6	Chronomics [UK] Saliva COVID-19 Test Launched	20 May 2020
7	Phosphorus gets FDA EUA for home saliva test [collection]	9 June 2020
8	Sysmex Japan gets approval in Japan with BGI RT-PCR Kit	10 June 2020
9	Yale/Saliva Direct working with the NBA	24 June 2020
10	CRL/OraSure get EUA with RT-PCR	31 July 2020
11	Approval for Yale/Saliva Direct	17 August 2020
12	U of Illinois-Urbana-Champaign FDA EUA approval granted	20 August 2020
13	OraSure/MiraDx get EUA for RT-PCR Kit	4 September 2020
14	Spectrum DNA gets FDA EUA for saliva collection kit	19 October 2020
15	DNA Genotek’s OMNIgene·ORAL OM-505 and OME-505 saliva collection devices receive FDA EUA	11 February 2020
16	AZOA P23 At-Home COVID-19 Home Saliva Collection Kit available at Costco Retail outlets for $129.99–$139.99	11 March 2020
17	PerkinElmer coronavirus RT-PCR assay receives CE Mark for saliva use, first for sample pooling	17 December 2020
18	Kleva Health’s at-home COVID-19 saliva test kit achieves EUA	12 September 2020
19	UAE: Scientists develop smartphone-read saliva testing method for Covid-19	12 October 2020
20	KnowNow COVID-19 saliva lateral flow from Vatic [UK] launched	19 October 2020

**Table 2:** Comparison of studies using saliva-based testing versus conventional swab-based testing for the detection of SARS-COV-2.

Study	Ref	Saliva Collection Method	Swabs and Lavage for Comparison	Diagnostic Test	N	TP	FP	FN	TN	Sensitivity	Specificity	PPV	NPV
Azzi, L et al., 2020 (Italy)	[12]	Drooling	NPS	RT-PCR	25	25	0	0	0	1	uc	1	uc
Azzi, L et al., 2020 (Italy)	[13]	Drooling	BAL	RT-PCR	2	0	2	0	0	uc	0	0	uc
Chen, Lili et al., 2020 (China)	[14]	Cotton Swabs—Saliva from orifices	OPS	RT-qPCR	31	4	0	9	18	0.31	1	1	0.66
Han, Mi Seon et al., 2020 (Korea)	[15]	Saliva	NPS, OPS	qPCR	2	1	0	1	0	0.50	uc	1	0
Wang, To et al., 2020 (Hong Kong, China)	[16]	Sputum/Coughed-out Saliva (self-collected)	NPS	RT-qPCR	12	11	0	1	0	0.92	uc	1	0
Wang, To et al., 2020 (Hong Kong, China)	[17]	Coughed-up Saliva—Posterior OroPharynx	NPS, Sputum	RT-qPCR	23	20	0	3	0	0.87	uc	1	0
Wyllie Anne et al., 2020 (USA)	[18]	Saliva (spitting)	NPS	rRT-PCR	46	38	1	7	0	0.84	0	0.97	0
Zheng Shufa et al., 2020 (China)	[19]	Sputum (hospitalized patients)	Stool, Serum, Urine	RT-qPCR	96	96	0	0	0	1	uc	1	uc
Zhang Wei et al., 2020 (China)	[20]	Oral Swabs (hospitalized patients—baseline)	Blood, Anal	RT-qPCR	16	8	0	8	0	0.50	uc	1	0
Pasomsub, E et al., 2020 (Thailand)	[21]	Saliva	NPS, TS	RT-PCR	200	16	2	3	179	0.84	0.98	0.88	0.98
Somrak et al., 2021	[22]	Self-collected	NPS	RT-PCR	32	12	0	20	0	0.37	1	1	0.91
Basso et al., 2021	[23]	Self-collected	NPS	RT-PCR	84	67	0	17	0	0.78	uc	1	0

Sample size (N); true positive (TP); false positive (FP); true negative (TN); false negative (FN); positive predictive value (PPV); negative predictive value (NPV); nasopharyngeal swab (NPS); broncho alveolar lavage (BAL); oropharyngeal swab (OPS); throat swab (TS); real-time reverse transcription polymerase chain reaction (rRT-PCR); reverse transcription polymerase chain reaction (RT-PCR); unable to calculate (uc). Sensitivity and specificity calculations were performed through an online tool (http://vassarstats.net/clin1.html) (accessed on 29 April 2021).
